# Understanding the usability issues in contact management of illiterate and semi-literate users

**DOI:** 10.1371/journal.pone.0259719

**Published:** 2021-12-02

**Authors:** Shamaila Hayat, Aimal Rextin, Anas Bilal

**Affiliations:** 1 Department of Computer Science, University of Poonch, Rawalakot, Azad Jammu and Kashmir, Pakistan; 2 Department of Computer Science, COMSATS University Islamabad, Islamabad, Pakistan; 3 Department of Computer Science, FAST National University of Computer and Emerging Sciences, Islamabad, Pakistan; Fuzhou University, CHINA

## Abstract

The effective utilization of a communication channel like calling a person involves two steps. The first step is storing the contact information of another user, and the second step is finding contact information to initiate a voice or text communication. However, the current smartphone interfaces for contact management are mainly textual; which leaves many emergent users at a severe disadvantage in using this most basic functionality to the fullest. Previous studies indicated that less-educated users adopt various coping strategies to store and identify contacts. However, all of these studies investigated the contact management issues of these users from a qualitative angle. Although qualitative or subjective investigations are very useful, they generally need to be augmented by a quantitative investigation for a comprehensive problem understanding. This work presents an exploratory study to identify the usability issues and coping strategies in contact management by emergent users; by using a mixture of qualitative and quantitative approaches. We identified coping strategies of the Pakistani population and the effectiveness of these strategies through a semi-structured qualitative study of 15 participants and a usability study of 9 participants, respectively. We then obtained logged data of 30 emergent and 30 traditional users, including contact-books and dual-channel (call and text messages) logs to infer a more detailed understanding; and to analyse the differences in the composition of contact-books of both user groups. The analysis of the log data confirmed problems that affect the emergent users’ communication behaviour due to the various difficulties they face in storing and searching contacts. Our findings revealed serious usability issues in current communication interfaces over smartphones. The emergent users were found to have smaller contact-books and preferred voice communication due to reading/writing difficulties. They also reported taking help from others for contact saving and text reading. The alternative contact management strategies adopted by our participants include: memorizing whole number or last few digits to recall important contacts; adding special character sequence with contact numbers for better recall; writing a contact from scratch rather than searching it in the phone-book; voice search; and use of recent call logs to redial a contact. The identified coping strategies of emergent users could aid the developers and designers to come up with solutions according to emergent users’ mental models and needs.

## Introduction

Information and communication technologies (ICTs) have traditionally been designed for office-environment for professional and educated people who are referred to as *traditional* users [1]. The rapid decrease in prices of ICTs artifacts over the past few decades trickled them down to poor and less educated people having diverse geographical and cultural backgrounds [1–3]. These are referred to as *emergent* users, and they are disadvantaged in terms of capabilities to access, learn, and operate ICT artifacts [1]. These users often belong to developing countries and have lower education and limited technology exposure.

Currently, smartphones have become a prevalent technology with 5 billion cell phone users worldwide [4] and 79% of these users belong to developing countries [5]. According to Pew Research Centre, at least 3 − *in* − 4 or more people own a smartphone in every developing country they surveyed. However, Middle Eastern emerging nations report a higher percentage of up to 80% of smartphone ownership [4]. Smartphones have drastically changed the communication practices of users by introducing various interaction mechanisms [6, 7]. Smartphone users own diverse cross-cultural social groups due to the proliferation of a multitude of social media applications [4]. Modern communication applications provide a variety of features [8–10] which have made them very popular among students [11], and people of all ages [10]. These features provide an easy way to convey emotions, behaviours, and attitudes in textual conversation [10], hence, making texts the most frequent communication channel in long-distance relationships [12]. However, emergent users were found to use voice communication mainly for maintaining their social connections [13].

Beyond communication, smartphones offer a variety of services such as income generation [14, 15], mobile banking, health management [16], entertainment [17] etc. Unfortunately, 781 million people across the world can not avail these services because they are illiterate and 85% of these belong to developing countries [16, 18, 19]. Regrettably, smartphones are primarily designed with the traditional users in mind, leaving the emergent users at a disadvantage in using smartphones. Many studies recommend that interfaces for emergent users should be designed after understanding their problems and needs [20, 21]. A number of new interfaces for emergent users were designed using this approach, one example is new interfaces for employment search and maps [22]. Other studies take a different approach and instead suggest that the emergent users might be better facilitated by allowing them to take live help from more experienced users [23]. However, this approach, is not scalable as it is not possible to provide live help to every emergent user whenever needed.

Despite their numerous functions, cellphones are still primarily used for communication; with text and calls being the most common communication channels [24–26]. Communication is initiated in these channels by first searching a contact in the call log or the contact-book. We denote tasks like saving a contact, searching a contact as *Contact management*. Both saving and searching a contact are difficult tasks for emergent users [20, 27]. However, new contacts are added occasionally, and hence, it is probably feasible to get help from a more experienced user, while searching for a contact is a frequent task and it is not practical for them to take expert’s help every time. Emergent users have adopted different strategies to help them search their required contacts. Some coping strategies studied in the literature include: writing phone numbers with different colours [20]; using the call log to select a recently called contact [27]; saving images with contacts [28]; maintaining a very short contact list and memorizing the last few digits of a number or adding special symbols like # in the contact names as indicators [27, 29]; entering a number from scratch each time [30].

In this study, we decided to explore the contact management coping strategies adopted by Pakistani emergent users. This is because Pakistan is a developing country, where 43% of adults (15 years or older) are illiterate [31]. In 2021, cell phone subscriptions in Pakistan have been reported to reach up to 185 million, which is almost 85% of the total population [32]. Whereas, almost 51 million people own smartphones [33]. We observed that emergent smartphone users encounter many issues while using smartphones, hence, there is a need to explore the general coping strategies and their effectiveness in the context of Pakistani smartphone users. Thus, the following research questions arise:

**RQ1:** What coping strategies for contact management are used by the emergent smartphone users of Pakistan and if they are effective?
**RQ2:** Given these contact management coping strategies, how is the composition of emergent users’ contact-book quantitatively different from those of traditional users?

### Goals and objectives

Hence, the main objectives of this study include the following:

- We provide a detailed exploration of coping strategies of Pakistani emergent users in contact management. Specifically, we will analyse whether all emergent users use the same coping techniques, or there are any differences across cultural boundaries by comparing our findings with the literature.
- We aim to explore the difference in the composition of contact-books of both emergent users and traditional users. We will quantitatively measure some important factors such as the total number of saved contacts, percentage of dialled numbers from contact-book, and proportion of unintelligible contact names in emergent users’ contact-books that were overlooked in previous studies. Note that if a contact number is saved using meaningless names such as letters, numbers, special symbols, or any combination of letters and symbols, etc., it is declared as an unintelligible contact name.
  A detailed exploration of these factors could help us to come up with a better contact book design for emergent users. For example, in the case of fewer unique contacts, an iconic contact list could easily resolve the contact saving and recall issue. Similarly, the proportion of unintelligible contact names and dialled numbers through the contact-book would reveal the preferred contact management strategies of these users, which could be utilized in future designs.

### Contributions

1. We identify major themes in coping strategies of Pakistani emergent smartphone users by conducting an interview-based study and then evaluate their effectiveness through a usability study.
2. We will answer the second research question by adopting a quantitative and programmatic approach and compare the log information of these two users groups to see how the composition of contact-book of emergent users differ from those of traditional users in aspect like its size and prevalence use of special symbols etc.
3. Similarly, we quantitatively explore several unanswered questions in past emergent user studies, like the average size of contact-book, the proportion of dialled numbers through contact-book, percentage of unintelligible contact names, etc.

Our findings confirmed that Pakistani emergent users also adopt many coping strategies as discussed in the literature. However, unlike previous findings, our participants didn’t prefer icons to recall a contact. Most users were unaware of quick access features provided in many phones like speed dial, google voice service to search information. The contact list size of emergent users was found much smaller than that of traditional users. Similarly, the proportion of unintelligible contact names of emergent users was found significantly greater, and their use of call logs or contact-books to redial a contact was much lower as compared to traditional users.

## Related work

Over the past few decades, better Internet and ICT services have brought revolutionary changes in global development. Since the 1990s, these services have changed technology from a tool, to how it can act as a central force in the economic and social development of nations [34]. The rapid decrease in the prices of ICT artifacts from PCs and laptops to handheld devices has made them accessible to low-income groups. These technologies have reformed the ways of production, trade, and delivery of traditional services [35]. For example, all traditional ways of managing health care, checking market updates, money transfer, management of emergency responses, communication with government officials, and many other things have changed after the upsurge of ICTs in society. Hence, authorities related to global development consider ICT as a potential platform for providing services related to global development [16]. However, the effective utilization of these technologies is more challenging in developing nations where more than two-thirds of the population is illiterate and many more who are classified as literate have great difficulty in reading and writing [36].

Along with literacy, cognitive abilities also play an important role in the adoption of ICT and internet-enabled devices such as mobiles, smart devices, and computers etc. These abilities have much importance in the learning process, as the use of different cognitive skills and techniques can enhance the learning process. Corrective feedback is an example of such behaviour in computer-based learning environments and it showed significant and quick learning, deep understanding, and the ability to transfer knowledge. In this context, different interaction modalities have been proposed by researchers. An example is C3STEM, a framework for improving computational thinking skills of STEM students. STEM stands for Science, Technology, Engineering, and Mathematics [37]. There is a significant connection between the level of education and the cognitive abilities of people [38].Research in the field of cognitive science has shown that people with limited formal education have different cognitive skills as compared to people with good formal education [39]. These skills include visual organization and visual memory, language processing, mental and spatial orientation, vigilance, speed of cognitive processing, divided attention, and perceived self-efficacy [40, 41]. Studies have shown that beyond the inability to read and write, illiteracy is also correlated with cognitive skills for example Medhi et al. showed that low-literate people have great difficulty when it comes to transfer learning in video-based skills training [42], and in the navigation of hierarchical structures of information [43]. Hence, illiterate users essentially have different user interface requirements than traditional users.

Furthermore, researchers have conducted several anthropological studies to understand the issues associated with technology usage and the underlying causes of failure of mobile and ICT initiatives in the developing countries [44, 45]. Sekakubo et al. conducted interviews with the people directly involved in the e-learning process with the help of learning management systems (LMS). They identified that high ICT illiteracy rates; low comfort levels with technology; usability issues of LMS; poor marketing strategies; ineffective maintenance strategies; and insufficient user/technical support etc., are the root causes of LMS failure in South African population [44]. Gitau et al. identified the barriers in mobile-centric internet use in a large under-developed and less-privileged community [45]. All of these findings ultimately help designers to improve the usability of ICT artifact for less-privileged population.

Human-computer interaction (HCI) has an important role in empowering users and adapting technology to local needs [46]. HCI researchers seek to improve the interaction between human and computer by improving the usability of interfaces [47]. They explore new methods for designing, implementing, evaluating, and comparing user interfaces concerning usability and other desired properties. HCI researchers have employed many input-output techniques other than text to increase the usability of traditional interfaces for emergent users. These techniques include the use of speech and touch for input [48, 49], graphical output [30], audio combined with graphics or text and Interactive Voice Response systems (IVR) [50–52], etc.

Smartphones have become ubiquitous in developing societies. They are mainly used for communication along with the provision of many other services. These devices have drastically changed the traditional phone usage [53]. Current smartphones offer various interaction mechanisms [6, 7] through a variety of communication applications; which offer many interesting features for conveying emotions in textual communication [8–10]. However, many users find these services difficult to use because 781 million people around the world are illiterate, and many more are barely able to read and write [16, 18]. Therefore, designing the same interfaces for users of different literacy levels and technology exposure is not justified as the skills and design needs of emergent users are very different from traditional users [20, 21]. However, designers seldom consider the needs of emergent users when designing ICT artifacts.

Existing studies have found that textual interfaces are less usable for emergent users [22] and hence, impose a psychological barrier in their usage [54]. Most emergent users were found to respond to texts by calling the sender [27] and they also preferred graphical interfaces [55]. Similarly, audio support along with graphics seemed to significantly increase usability [56]. Therefore, previous research suggests non-textual interfaces using icons, images, voice, colours, etc. for better understanding of emergent users [18]. Researchers have proposed a number of non-textual interface designs in many areas including financial management [57, 58], farming [48, 59], domestic labourers [22], health workers [49], contact management [54, 60, 61], tools for retrieving contact information for agriculture-related enterprises for low literate users [62], job searching sites [63], etc. Similarly, speech enabled input through keyboards [64, 65] and virtual keypads for local languages, seemed to help low-literate users in text entry [66]. Furthermore, Frid et al. [67] suggest adopting *Design for All* approach to make technological products usable for everyone. This approach focuses on some basic design principles to increase the usability of products such as simplicity, flexibility, and user involvement in design process, etc.

Emergent users mainly use smartphones for maintaining their social connection through voice communication. Usability and sociability are closely related [68], however, current smartphone interfaces have many usability issues. In these interfaces, contact management i.e., call initiation, saving, and searching a contact is hard [13] as it requires some level of literacy. Below, we summarize some studies that investigated contact management issues of emergent users or proposed solutions for them.

### Contact management issues of emergent users and proposed solutions

A comprehensive literature search revealed some studies which explored contact management and call initiation strategies of emergent users. All of these studies employed various subjective approaches including interviews, surveys, ethnographic studies, and a user-centred design approach to explore the usability of current textual interfaces for emergent users. Below, we briefly describe the techniques, number of participants, findings, suggestions, and proposals of each of these studies.

Bhamidipaty and Deepak surveyed 20 emergent users and found that participants used minimal phone features and had fewer contacts including family and close friends. Most users saved the numbers with the help of literate users and learned keystrokes to dial important contacts. This study proposes a symbol-based key-pad that allows users to save and search contacts by using a combination of available symbols [54].

Lalji et al. [20] and Friscira et al. [50] used a user-centred design approach through interviewing and prototyping to investigate the design needs of emergent users. The participants were found to adopt different strategies to recall contacts such as memorising important numbers and writing them on paper etc. The last dialled number was also found to be helpful in redialling contacts. They suggested using images, and voice tags with contacts. They also suggested providing a drawing area on the phone-book for user-created symbols with contacts.

Some researchers conducted interview-based studies [27, 60]. Knoche et al. [27] interviewed 9 illiterate users. Participants stored contacts on papers, business cards, and registers. Spatial locations, patterns, doodles, shapes of paper, the colour of ink, and paper were used to recall a contact. Many contacts were saved with the help of others and usually contained the first letter followed by special characters such as Nc#-, etc. In some cases, participants tried to recall a contact through salient features such as repetitions of digits, city or country codes, etc. Text containing the contact number was used as another way to lookup for contacts. Joshi et al. [60] interviewed 11 participants and found that most participants used paper diaries to save contact numbers and tried to remember important contacts by locations. Participants adopted various organization techniques such as saving contacts from same places at the same location, maintaining sequence by keeping older first, annotating contacts from the same family or relatives. Users saved 10 − 15 contacts on phone with the help of others. To keep the top contacts on the top and on the same location, digits were added with contact names e.g. 1 = Home, 2 = Aunt, etc. Users were unaware of various contact organization features available on phones. Also, some users used abbreviations to categorise users based on profession, location, and business. This study proposed a contact-book that organises up to 81 contacts on nine colour pages, having 9 icons on each page such that the location of each contact is always kept constant.

Ahmed et al. [23] explored that all participants of the study faced some difficulties in saving and searching contacts. They tried to memorize the contact numbers or the position of certain contacts in the contact list. Different symbols were used with each contact to assist in recalling them more easily. Hence, an interface was proposed so that they can seek remote help from expert users in placing calls and saving contacts.

**Literature gap**. All frequently used communication applications including WhatsApp, text, phone calls, etc., require the use of the contact-book for efficient contact management and retrieval. However, low literacy causes hurdles in contact management. The majority of emergent users are unable to make effective use of the contact-book. Hence, they adopt alternative strategies to store and retrieve contact numbers such as memorizing contacts [20]; placing contacts at different locations; dialling a number from scratch each time [30], etc.

A comprehensive literature search revealed only a few studies exploring the contact management problem of emergent users [13, 20, 23, 27, 50, 54, 60, 69]. None of these studies explored call/text log data of emergent users to explore the quantitative differences in the composition of emergent users’ contact-books from that of traditional users. Similarly, the effectiveness of coping strategies adopted by emergent users to search and dial a contact was not measured in the existing literature. Therefore, this paper explores these factors by using a mixed approach.

## Current Pakistani users’ contact-book management techniques and their effectiveness

We were not sure if the coping strategies discussed in the literature also apply to the Pakistani population. Hence, we first tried to identify their coping strategies through a semi-structured qualitative study and then evaluated their effectiveness through a usability study. We will discuss them one by one below.

### Interview study

Interviews of participants are routinely done in HCI studies to get a preliminary understanding of the user behaviour and problems [10, 20, 50]. A semi-structured interview uses a mixture of open-ended and closed-ended questions followed by why or how questions. These are found useful in mixed-method research where you want to conduct a preliminary analysis before designing your overall research strategy [70], and have been used in many research studies [10, 20, 50]. Hence, our purpose of choosing an interview-based study was to get a preliminary understanding of the emergent users’ contact management strategies. We will briefly explain our participants recruitment criteria, protocol of interview process, and interview findings in the following sections.

#### Participants recruitment

We recruited 15 low-literate participants from Islamabad and Kashmir who were willing to share their experiences and also let us look at their contact-books. We used purposive sampling for selecting our user group, i.e. the participants were selected to cover low literacy smartphone users of both genders. Out of total participants 7 were sweepers at a local university in Islamabad; 5 housewives, 2 house keepers, while 1 was a peon.

#### Privacy and personal information

The first author explained the purpose of the study before data collection. All of the participants were informed that their data will be used only for research purpose and only those participants were recruited who participated on voluntarily basis. The personal information of participants like name and contact number were kept anonymous due to privacy concerns. However, the authors collected their gender, age, profession, education, and the contact management applications’ usage period information.

#### Demographics

The age of the participants ranged between 15 − 45 with the education level of all participants below grade 8. We note that $\frac{13}{15}$ participants had difficulty in reading and writing, further information about these participants is given in Table 1.

**Table 1:** Demographics of semi-structured qualitative study participants.

Participants	Age	Gender	Education	Usage Period	Profession	Place
1	40	M	Nil	4 months	Sweeper	Islamabad
2	28	M	7	5 years	Sweeper	Islamabad
3	36	M	5	2 years	Sweeper	Islamabad
4	30	M	7	3 years	Sweeper	Islamabad
5	23	M	6	4 years	Sweeper	Islamabad
6	30	M	5	10 years	Sweeper	Islamabad
7	34	F	5	1 year	Sweeper	Islamabad
8	40	M	7	6 years	Peon	Islamabad
9	23	M	Nil	8 years	Housekeeper	Islamabad
10	35	M	Nil	4 years	Housekeeper	Kashmir
11	20	F	Nil	2 years	Housewife	Kashmir
12	15	F	Nil	2 years	Housewife	Kashmir
13	42	F	3	1 year	Housewife	Kashmir
14	45	F	Nil	2 years	Housewife	Kashmir
15	22	F	Nil	3 years	Housewife	Kashmir

#### Interview method

The first author conducted individual discussion sessions of 10 to 15 minutes with each of the participants and prepared written notes of all of the participants’ responses. The notes were later, cross-checked and analysed by the second and third authors to deduce the results. Note that the author prepared separate coloured notes (sticky notes) for different responses. All the interview notes are available along with the demographics information of the participants. The time and location of the interview were decided based on the convenience of the participants but we ensured that it was a quiet place without any distraction. Our main questions are given in Table 2, follow up questions were asked if any point needed further investigation. The main interview findings are briefly described in the incoming sections.

**Table 2:** Questions explored during each interview session.

Question No.	Questions
1	How old are you?
2	What is your education?
3	How long have you been using smartphone?
4	What is the main purpose of using smartphone?
5	Do you have any difficulty in phone usage?
6	Do you have any difficulty in saving, searching and dialling a contact?
7	Do you take help from others to save a contact?
8	How do you save a contact by yourself?
9	Do you prefer text or voice call?
10	How do you dial a desired contact if you cannot read names?

We would also like to add that all the participants were verbally informed about the purpose and objectives of the study, hence, it was an informed consent. Only the participants willing to participate and share their information and phone data were recruited. They were allowed to leave the study any time they feel uncomfortable or they are reluctant to share their data. The Departmental Ethics Committee of COMSATS University, Islamabad reviewed the study protocol and confirmed that this study adheres to required ethical standards. The approval letter was numbered on 22-11-2019/CS/ETHICS. In all parts of the study, consent was taken verbally and recorded in the administrative file of this project. Their personal information was not used in the analysis of the results of this paper in any way. The reason written content was taken was that most of our participants were emergent users who had severe reading and writing difficulties, making written consent impractical.

#### Qualitative data analysis

**Fig 1:**
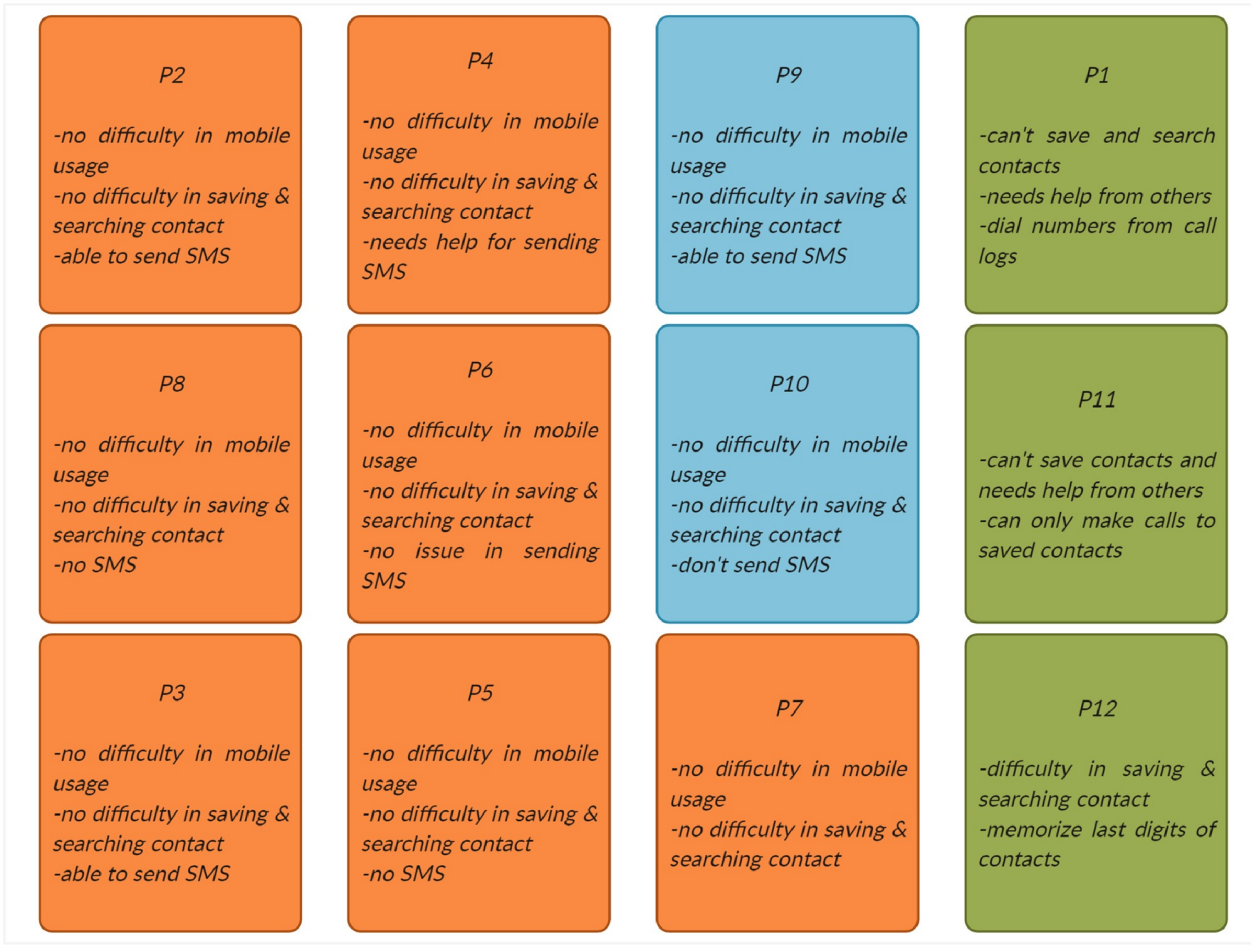
These affinity walls showing the results of our user study. Different emerging themes are shown with the help of different colours. Note that the demographic information of all study participants is given in Table 1.

*Affinity wall and thematic analysis*. The authors prepared an affinity wall based on all the responses of the interviews and organized all the responses into different emerging themes as shown in Fig 1. The authors then performed a thematic analysis and the following three major themes emerged.

- Participants having education level equal to grade 5 − 7, had no difficulty in using mobile phones and contact management applications.
- Participants with no education had difficulty in using contact management applications.
- Completely illiterate participants who had no difficulty in using contact management applications were found to use advance features for searching and saving a contact such as voice search etc.

*Interview findings*. Note that, all of the study participants owned their own smartphones but interestingly, two female participants used the smartphones of a male relative like their husband or a brother. The major interview finding are given below.

- The participants used smartphones for various purposes including social media usage, communication, taking pictures, and entertainment. All participants preferred voice communication including phone calls, WhatsApp voice, and voice notes rather than text messages due to reading and writing difficulties. Most of our participants also reported taking help to read text messages.
- The interviewer explored the participants’ call logs and contact-books with their consent to find the average contact-book size and contact saving strategies. Interestingly, we found that their contact-book size was much smaller than the average size of more than 300 contacts for traditional users [71]. More specifically, the size of participants’ contact-books ranged between 5 − 47 contacts with many repeated entries. The mean number of contacts was 24.3 while the standard deviation was 14.8. Participants reported taking the help of others to save contacts and when no help was available they tended to use random characters.
- Apart from one participant none was aware of the google voice facility to save and search contact numbers. Note that, this participant was completely illiterate but he had no difficulty in contact management due to the adaptation of google voice facility. Similarly, none of the participants were aware of the speed dial facility and instead used various coping strategies to search and recognize a contact. These strategies include memorizing the whole number or last few digits, adding a special sequence of characters with the name to help them recognize it. Most participants did not prefer icons with contact numbers as they usually forget what icon was used with a particular contact number when the number of contacts increases. However, some of our participants preferred real pictures and some suggested that icons closer to real pictures of participants could help them better to recall a number. Most participants claimed to have memorized important contact numbers and entered the number from scratch each time they want to call them. Another popular strategy that we noted was relying on recent call logs to identify people they contacted recently.

Based upon the above findings we decided to evaluate the usability of contact management applications (phone-book, call logs) for illiterate users which is discussed in the next section.

### Usability of current contact-books and call logs by emergent users

We now discuss the usability of the current textual contact-book interface by evaluating it with emergent users. Usability analysis evaluates the performance of a product by testing it with real users. Usability is a combination of three factors: effectiveness i.e. task accuracy; efficiency i.e. time taken to complete a task; and the satisfaction which indicates the user’s liking or disliking of the system [72, 73]. The participants, method, and results of this analysis are discussed below.

#### Participants’ recruitment

We recruited 9 completely illiterate participants including 2 females and 7 males, from Rawalpindi and Islamabad. We employed purposive sampling for participant selection, i.e. the participants were selected to cover low literacy smartphone users of both genders. Only those participants were recruited who were willing to participate in the study and let us look at their logs. These participants included 2 housewives, 6 greengrocers, and 1 housekeeper. Their age ranged between 17 to 50 years, and *they all used smartphones on daily basis*.

#### Method

The usability analysis of the call log and contact-book interfaces was conducted individually with each participant. We contacted different participants according to their convenience during working hours and each participant was assigned a particular time slot i.e., on average 15 minutes. The usability study took 2 days to complete and the participants were free after their scheduled slot.

*Task*. The participants were assigned a single task of searching for a particular contact through the call logs or contact-books. All participants were asked to search for 4 of their contacts on their phones. Contact search tasks were divided into two categories: complex and simple. Frequently called contacts belonged to the simple category and infrequently called contacts were considered as complex tasks. We identified frequent and infrequent contacts by asking the participants about whom they frequently and infrequently communicate. The first author then wrote these down and randomly picked two contacts from each category. We cross-checked these contacts by noting their calling frequency from call logs.

#### Performance evaluation

As we have already discussed that *usability* is a combination of three factors: effectiveness, efficiency, and satisfaction [72, 73]. Hence, the first author evaluated the participants’ performance during the usability study in terms of efficiency, effectiveness, and satisfaction. Note that a limitation of our usability study is that it was all done by the first author and other authors were not involved in independently verifying the results. Furthermore, generalizability was established by selecting our participants from different areas belonging to various professions and from both genders. In our scenario, the usability components were measured in the following way:

**Efficiency** was measured by noting down the total time (in seconds) spent searching for each contact. The time started when the first author read out the contact name to be searched and the participants started their search. The time ended when the user dialled a contact.
**Effectiveness** was measured by noting down the total number of errors. Here, if the dialled contact was not the same as the name given for search, it was an error and vice versa.
**Satisfaction** was measured by asking the users about their subjective opinion of the traditional contact-book and call log interfaces at the end of the study. The participants were asked a single question about their experience as done in [73, 74].

The validity of controlled experiments always has threats due to the impact of various confounding variables. The effect of uncontrolled variations was reduced by conducting the test at quiet places and at times when the participants were mentally relaxed. The participants used their own cell phones as they were only familiar with their own contacts, hence, interface learning difficulty was avoided. The point to be noted here is that the participants in the selected sample were completely illiterate between the age range of 17 and 50. Hence, the generalizability of the results to other groups of emergent users might not be so accurate. The results of the controlled experiment are briefly discussed below.

#### Results

**Fig 2:**
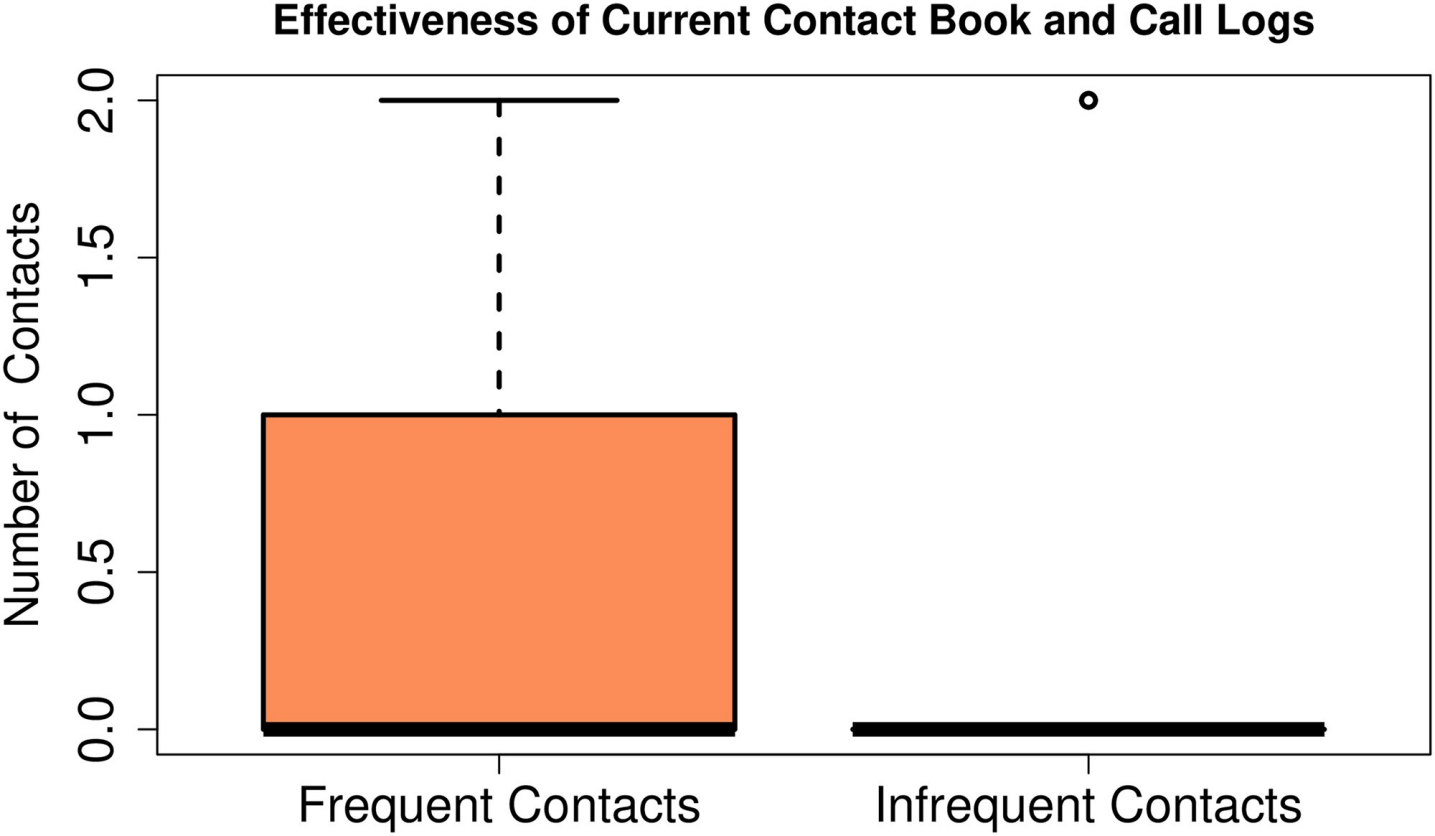
Effectiveness of the current contact-book and call log interfaces. This figure shows the number of correctly identified frequent and infrequent contacts through the traditional call logs and contact-book interfaces by illiterate users. It can be clearly seen that most users were unable to correctly identify both of their frequent contacts and none of them could find the infrequent numbers except one participant. This indicated the serious usability problem in the current smartphone interfaces for illiterate users.

First of all, the effectiveness of the traditional contact-book or call logs was analysed. More specifically, the number of errors made by the users while searching for the desired contact were counted. Interestingly, 2 out of 9 participants had no contacts saved in the contact-book. One of them was found to write down the contacts on a small diary with different colours or symbols to recall them easily. We noted that all participants except two used call logs to search for a contact. One of these two users had only 4 contacts saved by another literate user, the other had 6 contacts in his contact-book. Note that none of the participants had more than 20 saved contacts, with some repeated entries. Frequent contacts in all cases included close family members, whereas infrequent contacts included extended family and friends. All participants except four, identified all frequent contacts correctly but none could identify infrequent contacts except one. Note that 4 out of 9 users abandoned searching the infrequent contacts after a few seconds and their search was considered incorrect. Fig 2 shows the number of errors while searching frequent and infrequent contacts. Here it is clearly seen that almost all users successfully searched the frequent contacts but none were able to locate the infrequent contacts except one.

Secondly, efficiency was measured, i.e. the time spent by the users while searching for a particular contact. The study participants spent a mean time of 28 seconds with a standard deviation of 19.8 to search frequent contacts. As previously discussed, 4 out of total participants abandoned searching for the infrequent contacts after few seconds, hence, their search was not included in the final results. Thus, the mean time spent on searching for infrequent contacts of the remaining 5 participants came out to be 22.5 seconds with a standard deviation of 14.4.

**Fig 3:**
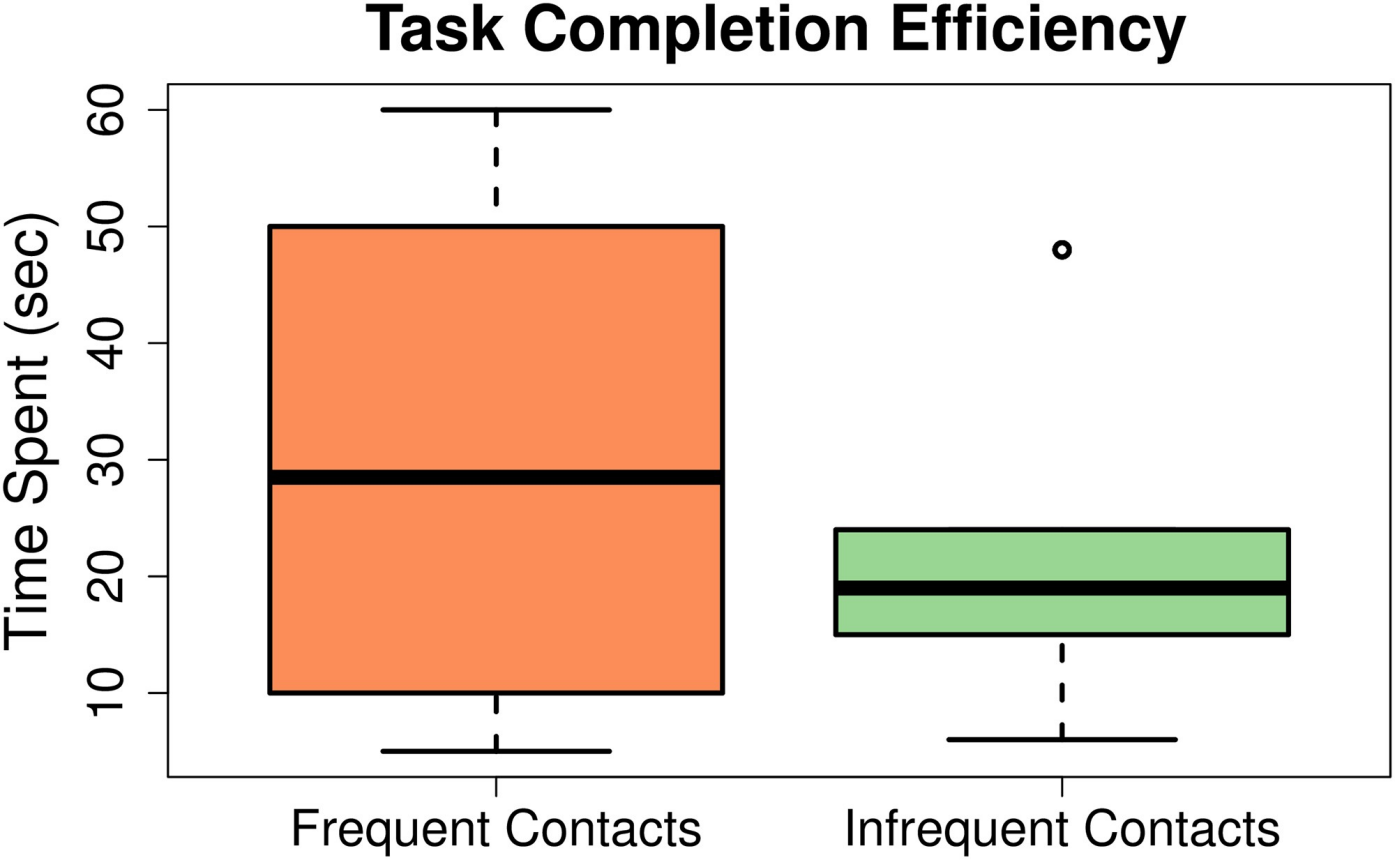
Contact search efficiency on traditional interfaces by illiterate users. As indicated by the figure, the time taken to search frequent contacts is higher than that of infrequent contacts. The reason is that, most users could not recall an infrequent contact hence, they randomly just dialled any contact that believed as the intended contact without putting more effort.

**Fig 4:**
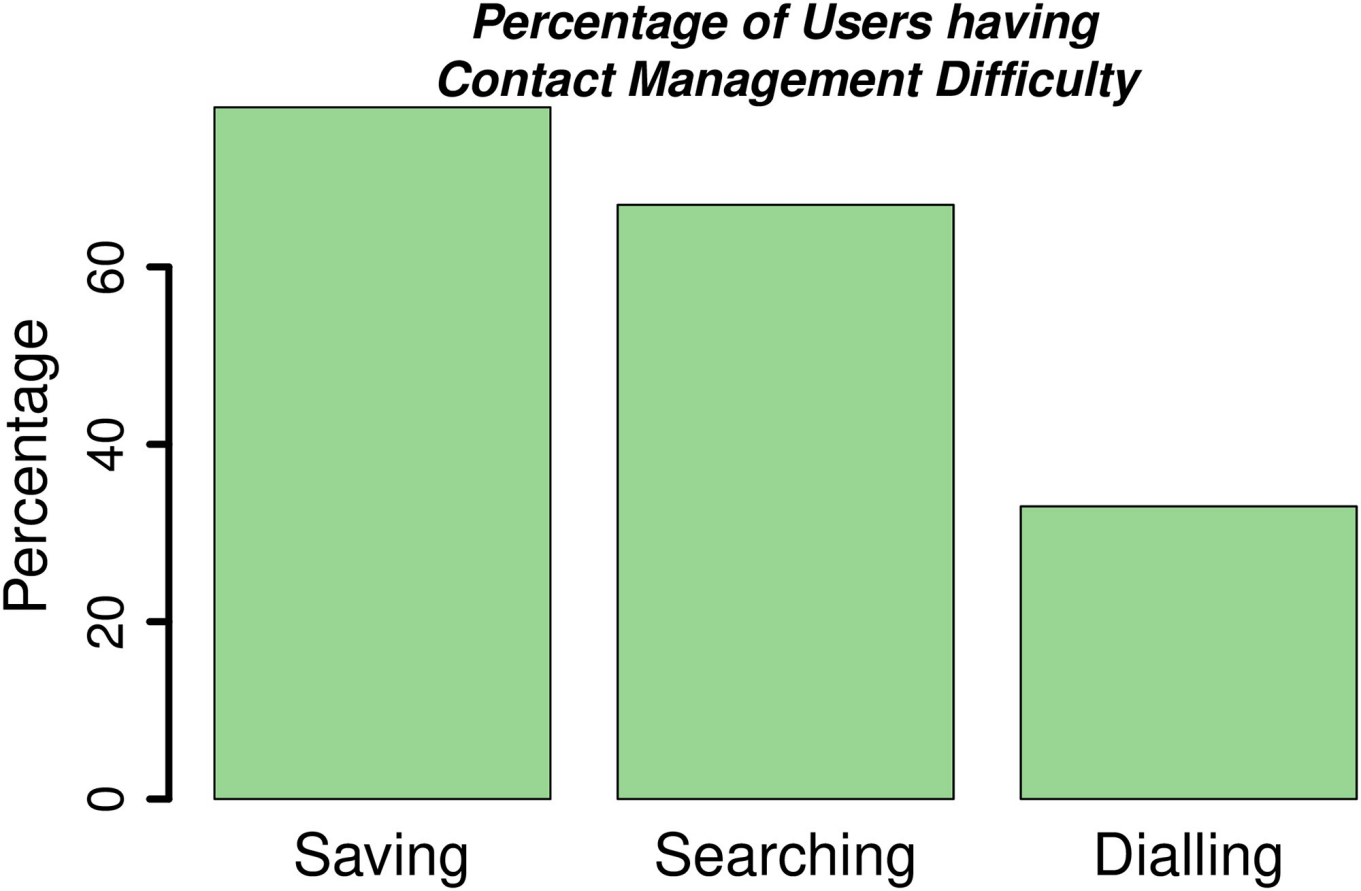
Subjective responses of emergent users about the usability of current contact-book and call log interface. It can be clearly seen that almost 80% users had difficulty in saving a contact, almost 70% in contact search, and more than 30% also had difficulty in dialling a contact number. Hence, the subjective responses of controlled experiment’s participants also indicate the severity of usability issues in the contact management on traditional smartphone interface, by illiterate users.

Fig 3 gives the overall pattern of time spent on searching for both types of contacts. Here, it can be clearly seen that the mean time spent searching for frequent contacts was higher than the mean time spent on searching for infrequent contacts. The reason behind the lower search time for infrequent contacts is that the users could not recall the infrequent contacts and they just randomly selected any contacts without exerting any effort. At the end of the usability study, the participants were asked a single question about their experience with the traditional contact-book and call log interface. We captured the overall user satisfaction of the traditional contact-book and call logs through a single question as recommended by Hornbaek [75] and used in [72, 73]. The question was, “*Do the users find it difficult to save, search, and dial a contact on the current smartphone interface?*” Fig 4 briefly summarizes the participants’ subjective responses.

Out of the total, 78% of the participants found it difficult to save a new contact as they were unable to write the names, hence, they relied on more experienced users to save the contacts. The remaining 22% claimed to save the contact by themselves but they were unable to write the names, hence, they used various symbols, letters, digits, or any combinations of these to recall a contact. Similarly, 67% of users found it hard to search for a particular number and preferred re-entering the contact from scratch. Finally, 33% of users were unable to even re-enter a contact, however, they were able to recall the last received or dialled number and redial it from the call log.

The call logs analysis of emergent users, interview findings, and the controlled experiment revealed that emergent users have severe usability issues. They face difficulties in saving, searching, and dialling contacts from the contact-book or call logs. Inspired by these findings we decided to further explore the contact management behaviour of emergent users by analysing dual-channel communication logs and contact-books of emergent users. Hence, a larger number of participants were recruited for data analysis. The user demographics, dataset characteristics, and results of this analysis are discussed in the next section.

## Objective data analysis of emergent and traditional users

This section provides a comparative analysis of dual-channel logs and contact-books of two user groups i.e. traditional users and emergent users. The purpose of this comparison is to highlight the contact management issues of emergent users more clearly.

### Participants’ recruitment

We recruited a group of 30 emergent users and a group of 30 traditional users from Islamabad and Kashmir. These participants were briefly introduced to the purpose and objectives of the study. Only those users who were voluntarily willing to share their communication logs and contact-books were recruited. We took their formal consent before collecting their communication logs and also showed them their extracted data files to ensure they are comfortable with sharing this data.

### Demographics

The age of participants in emergent group ranged between 17 − 56 years including 2 females and 28 males. A small number of participants had formal education (none above grade 8), however, they were still either completely unable or hardly able to read and write. All participants belonged to low earning professions including, construction workers, cleaners, drivers, cooks, etc., and had a monthly income between $60 to $190. However, a smaller proportion of emergent users (4 users including three small business owners, and one contractor) had better monthly income roughly ranging between $400 to $600 with possible fluctuations each month.

The traditional users comprised of 22 males and 8 females between the age of 26 − 45 years. These users belonged to various professions including, IT specialists, teachers, and software developers. The monthly income of these users ranged between $400 to $1223.

### Data collection and analysis method

To answer the second research question, we adopted a quantitative and programmatic approach and compared the log information of both user groups. Contact books and dual-channel (call, text) logs were collected from both user groups by using a customised Android application. The contact-book logs consisted of each contact’s name, number, and total contacts of the user. While the call and text logs contained the following information for each communication event: contact name/number, time, date, duration, and status (incoming, outgoing, missed).

We used R as a programming tool to see how the composition of the contact-book of emergent users differ from those of traditional users in aspects like its size, prevalence use of special symbols, the proportion of dialled contacts through the phone-book, and percentage of unintelligible contact names, etc. We further performed statistical analysis to verify that the phone-book composition of emergent users is significantly different from that of traditional users. Note that our data seemed to fit for t-test but it was not normally distributed hence, we applied non-parametric equivalent of t-test i.e. Wilcoxon Sign-Rank test. Before starting our analysis we performed the necessary data cleaning as given below.

#### Data cleaning

All data analysis studies require data cleaning before analysing it to avoid misleading results. Hence, as a first step, the datasets were cleaned to maintain data integrity. In the contact-books of users, we noticed that the same numbers were stored multiple times in different formats. Let suppose a contact number is “3335280228”, it can be stored as follows: “03335280228”, “+923335280228”, or “3335280228”. In order to avoid inconsistencies in the data, all contact numbers were converted in the same format by removing white spaces, + 92, and initial 0, in dual-channel logs and contact-books of all users.

The call logs of emergent users originally had 21, 338 calls. After removing erroneous records (i.e. incomplete records with missing time series values.), the emergent users’ dataset left with 20, 428 calls. The traditional users originally had 34, 875 calling events which came out to 34, 551 calls after cleaning. Table 3 shows the main statistics of emergent and traditional users’ call logs and contact-books. The calling data i.e. total contacts, user pairs, and outgoing calls of emergent users are quite low as compared to that of traditional users.

**Table 3:** Summary of main features of emergent and traditional users’ call logs and contact-books. Emergent users had a mean number of 100.9 (s.d 94) contacts which is quite low as compared to traditional users’ mean contacts i.e. 609 (s.d 385). The mean number of outgoing calls i.e. 337 (s.d 627) of emergent users was almost half of the traditional users’ mean number of outgoing calls i.e. 670 (s.d 434). Similarly, emergent users’ had a lower mean number of user pairs i.e. 73 (s.d 53) as compared to that of traditional users i.e. 1369 (s.d 79).

	Total		Mean		SD	
	Emergent	Traditional	Emergent	Traditional	Emergent	Traditional
Contacts	2826	18280	100.9	609	93.9	385
Calls	20428	34551	681	1152	1398	738
Incoming	6163	8937	205	343.7	547	263.1
Outgoing	10111	20092	337	670	627	434
Missed	4154	4302	138	143.8	259	102
Pairs	2182	4088	73	1369	58	79

The text logs of emergent users initially had 2604 texts and traditional users had 1, 83, 987 text records. The unsolicited text messages such as texts from marketing companies were separated. For this purpose, the contact numbers of all outgoing texts were considered as solicited contacts. In case of incoming text messages, the existence of incoming text numbers was checked in contact-books and call logs of respective participants. If the entry of an incoming text contact was found in the above mentioned logs, it was declared as solicited text and vice versa. After this filtration procedure, the emergent users were left with a total of 290 solicited texts which is much less as compared to solicited text records of traditional users i.e. 1, 57, 409 texts. Table 4 briefly summarises the properties of text logs of both user groups. These statistics clearly indicate the impracticality of textual interfaces for emergent users.

**Table 4:** Summary of main features of emergent and traditional user’ SMS logs including total number of texts, incoming texts, out going texts, total solicited texts, and the total number of unsolicited texts. Emergent users had a mean number of 87 (s.d 186) total text records including 10 (s.d 16) solicited texts. Whereas, traditional users had a mean number of 6133 (s.d 8895) total texts including a mean number of 5247 (s.d 7769) solicited text messages. Similarly, the mean number of outgoing texts of emergent users came out to be 2 (s.d 6) which is very low as compared to that of traditional users i.e. 2601 (s.d 3730).

	Total		Mean		SD	
	Emergent	Traditional	Emergent	Traditional	Emergent	Traditional
SMS	2604	183987	87	6133	186	8895
Incoming	2541	110808	85	3694	183	5541
Outgoing	63	78030	2	2601	6	3730
Solicited	290	157409	10	5247	16	7769
Unsolicited	2314	26578	77	886	179	1920

The findings of this analysis are discussed below.

### Results and data analysis

A comparison of the total number of saved contacts; the percentage of unintelligible contact names; and the proportion of dialled numbers from contact-books of both user groups is given below.

**Fig 5:**
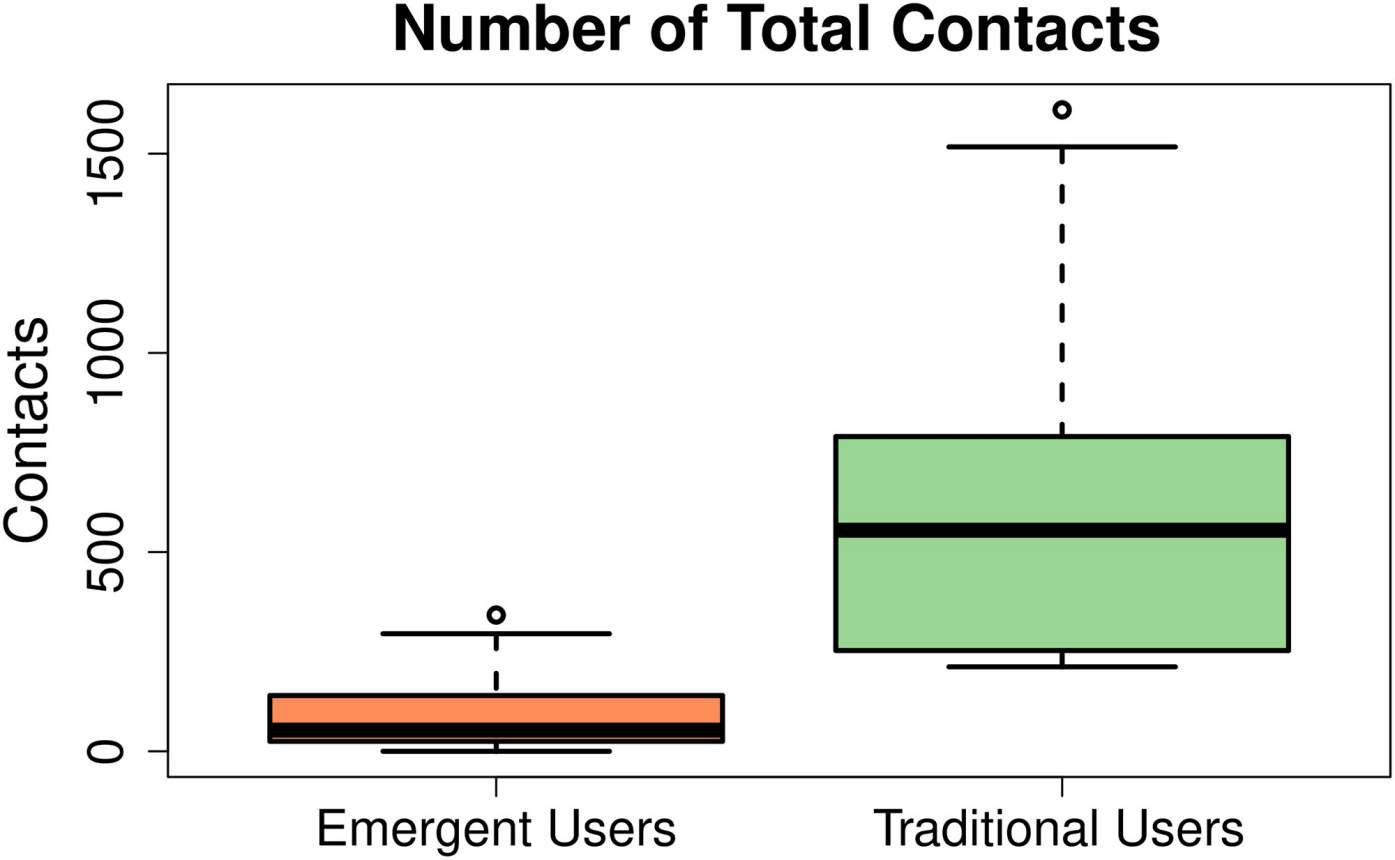
Comparison of total number of contacts between traditional and emergent smartphone users. Emergent users had very few number of total contacts as compared to traditional user. The mean number of emergent users’ contacts came out to be 100.9% (s.d 94%) whereas, traditional users had a mean number of 609% (s.d 385%) contacts.

**Total saved contacts.** The contact-books of all participants were analysed to compute the total number of saved contacts through a computer program. Fig 5 shows the total number of contacts saved in the phone-books of both user groups. Here it can be clearly seen that the emergent users had a much lower number of total contacts as compared to traditional users. More specifically, the mean number of total contacts of emergent users turned out to be 100.9 contacts with a standard deviation of 94. Whereas, the traditional users had a mean number of 609 contacts with a standard deviation of 385. Interestingly, the contact-books of 19.76% emergent users were empty which indicated an extreme usability issue in the current contact-book interface.

Here, we also noted that the participants in our emergent users’ group with little reading writing skills or better income resources had comparatively larger contact-book sizes. In fact, 6 participants including three small business owners, one contractor, one electrician, and one sweeper with the education level of grade 6, had larger contacts between the range of 158 − 342.

To analyse the statistical significance of our findings, we tested the hypothesis that the size of emergent users’ contact-book is smaller than that of traditional users. We formally state the hypothesis as follows: The null hypothesis (H_0_) is that the mean number of total contacts of emergent users (*μ*′) is the same as that of traditional users (*μ*), i.e. *μ* = *μ*′. Whereas, the alternative hypothesis (H_A_) states that the mean number of total contacts of emergent users is less than that of traditional users. We can mathematically write it as follows:

H_0_: *μ* = *μ*′
H_A_: *μ* > *μ*′

We applied Wilcoxon Sign-Rank test with 95% confidence interval and got a p-value < 3.05 × 10^−10^, indicating that the mean number of total contacts of emergent users is indeed much smaller than that of traditional users.

**Proportion of unintelligible contact names.** The findings of the interview-based study revealed that emergent users had great difficulty while saving contact numbers by themselves due to reading writing difficulties and sometimes they save contacts by themselves using meaningless names. For example, if a number is stored with A, or 12$, etc., it does not represent a proper name. We denote the contacts saved with such names as *unintelligible contacts*. The unintelligible contact names of emergent users were stored by using various combinations of alphanumeric characters and symbols that made no sense. These combinations included:

- random letters
- letters and digits
- digits
- blank entries
- special characters
- letters or numbers followed by special characters

**Fig 6:**
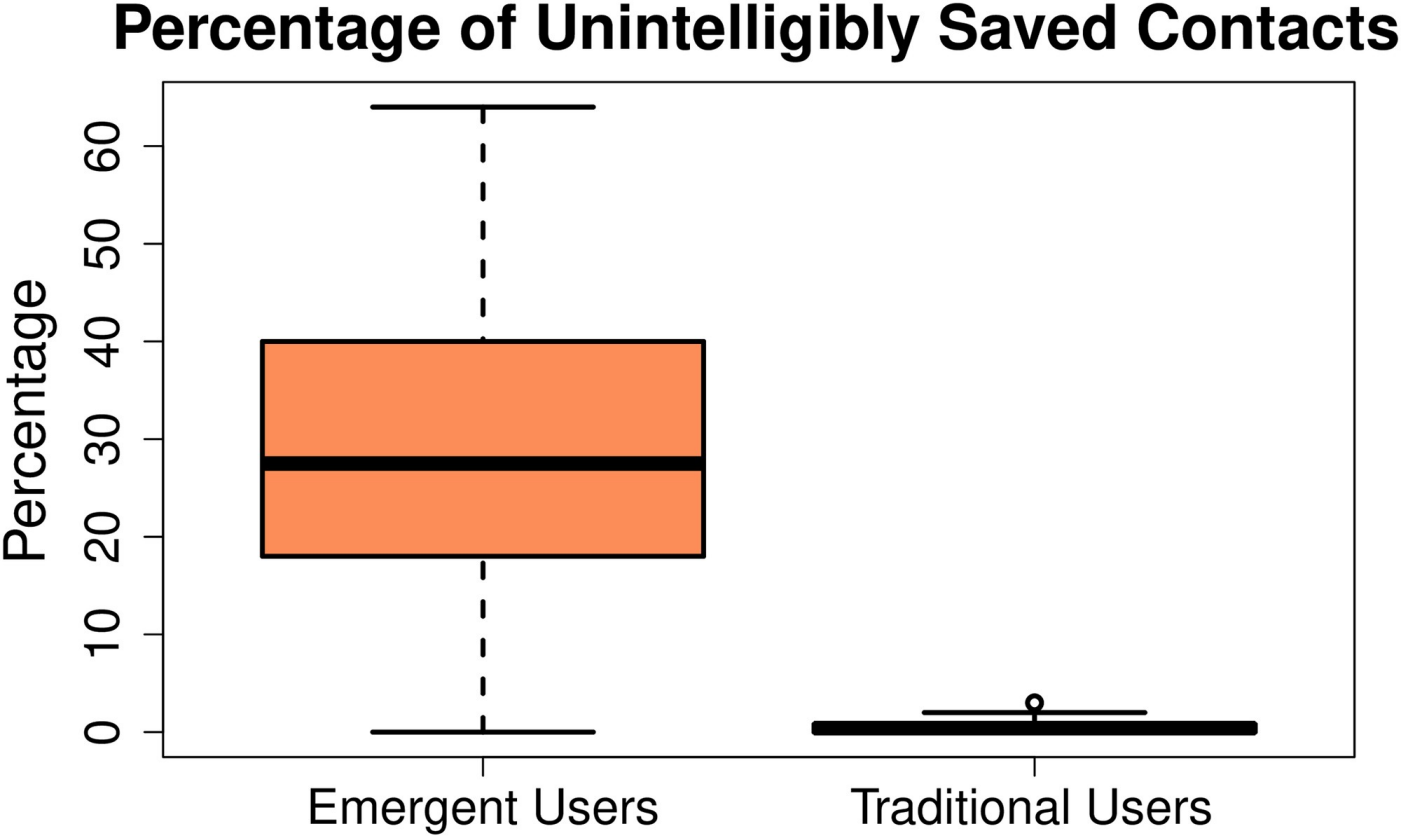
The contact-books of emergent users had a large number of contacts which were not saved using any meaningful names. The mean percentage of unintelligible contact names of emergent users came out to be 35.5% (s.d 23.6%) whereas, traditional users only had a negligible number of unintelligible contact names with the mean percentage of 0.48% (s.d.65%).

We computed the proportion of unintelligible contacts in both user groups. This was done by the first author who inspected the data files of each user on a computer one by one. The results were cross-validated by repeating the process three times on different days. Fig 6 shows the percentage of unintelligible contact names in the contact-books of both user groups. It can be clearly noticed that a significant proportion of contacts in the emergent users’ group was saved unintelligibly. In fact, the mean percentage of unintelligible contact names of emergent users came out to be 35.5% with a standard deviation of 23.6%. Whereas, in traditional users’ data this percentage was very low with the mean value of 0.48% unintelligible contact names and a standard deviation of 0.65.

We further tested the statistical significance of our findings by testing the hypothesis that the proportion of unintelligible contact names in emergent users’ contact-book is greater than that of traditional users. We formally state the hypothesis as follows:

The null hypothesis (H_0_) is that the mean number of unintelligible contact names of emergent users (*μ*′) and traditional users (*μ*) are the same, i.e. *μ* = *μ*′. Whereas, the alternative hypothesis (H_A_) states that the mean number of unintelligible contact names of emergent users is more than that of traditional users. We can mathematically write it as follows:

H_0_: *μ* = *μ*′
H_A_: *μ* < *μ*′

We applied Wilcoxon Sign-Rank test with 95% confidence interval and got a p-value < 1.311 × 10^−09^, which is much smaller than 0.05, so we can reject the null hypothesis and say with a high degree of confidence that emergent users have a greater proportion of unintelligible contact names as compared to that of traditional users.

**Proportion of dialled numbers through contact-book.** As previously discussed, the interview-based study participants revealed that they usually re-enter a contact from scratch each time, rather than using the contact-book. Hence, the call logs of both user groups were explored to find the percentage of outgoing numbers that were dialled from the contact-book. Note that if an outgoing number was present in the contact-book then it was assumed that it was dialled through the contact-book and vice versa.

To compute the proportion of dialled contacts via contact-book, the outgoing numbers were separated from the call logs of each user in both groups. Then the entry of each outgoing number was programmatically searched in the contact-book of a participant. If the entry was present, the contact was considered to be dialled using the contact-book. In this way, all outgoing contacts that were dialled using the contact-book were found for each participant and their proportion was computed for both user groups.

**Fig 7:**
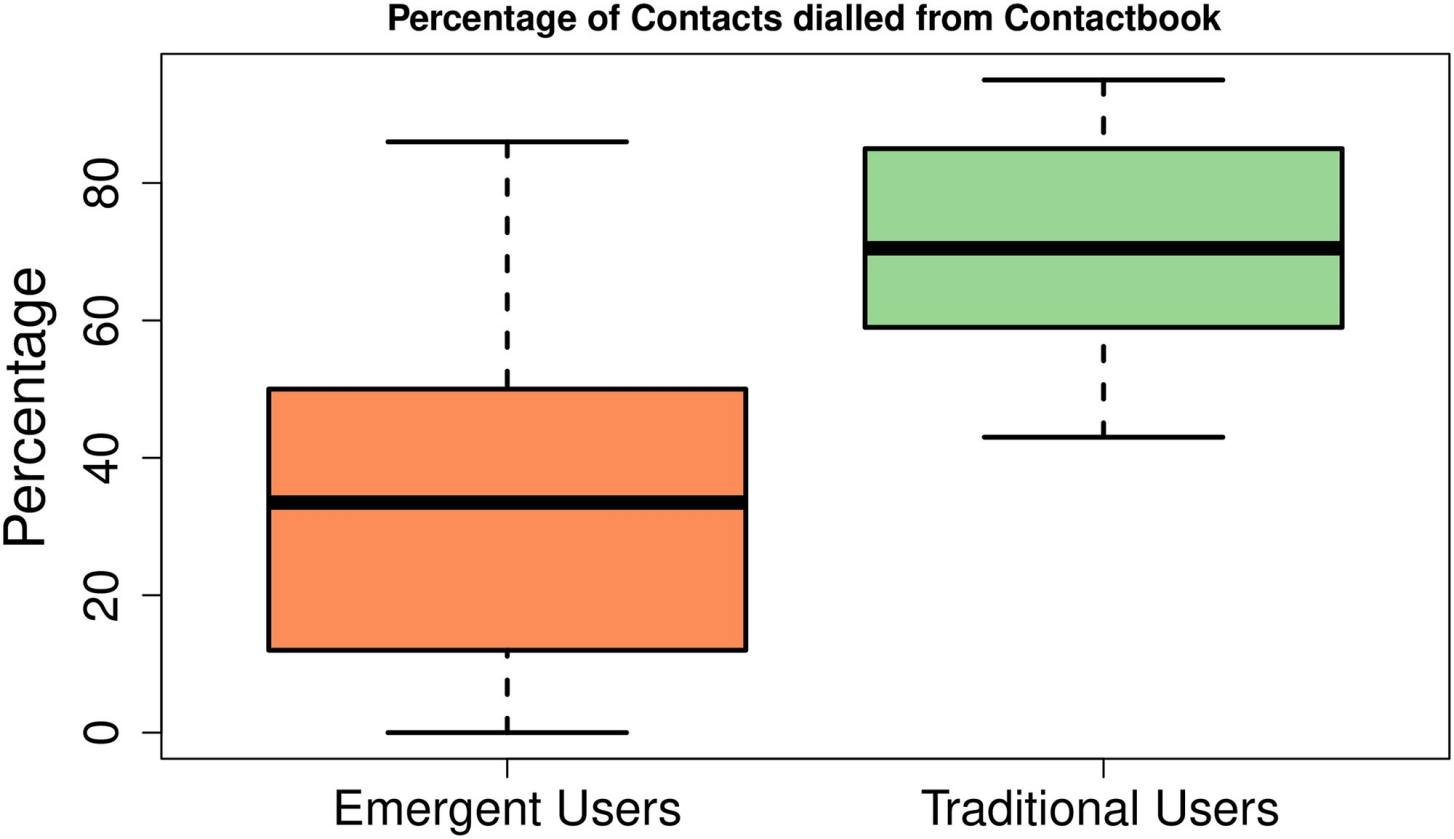
The mean percentage of dialled numbers through the contact-book in both user groups. Emergent users were found to dial fewer number of contacts through the contact-book as compared to traditional users. More specifically the mean percentage of dialled numbers through the contact-book of emergent users came out to be 35% (s.d 24.5%) whereas, in case of traditional users this percentage was 71% (s.d 15.6%) contacts.

Fig 7 shows the percentage of dialled numbers that were already saved in the contact-books of users. Here, it can be seen that a major proportion of dialled numbers by emergent users was not saved in the contact-book as compared to traditional users. More specifically, for emergent users, the mean percentage of dialled numbers through the contact-books came out to be 35% with a standard deviation of 24.5% whereas, the mean percentage of dialled numbers through the contact-books of traditional users came out to be 71% with a standard deviation of 15.6%.

We further tested the hypothesis that the proportion of dialled contacts of emergent users through the contact-books is less than that of traditional users. The null hypothesis (H_0_) is that the mean number of emergent users’ dialled contacts (*μ*′) through the contact-books is the same as that of traditional users (*μ*), i.e. *μ* = *μ*′. Whereas, the alternative hypothesis (H_A_) states that the mean number of emergent users’ dialled contacts through the contact-books is less than that of traditional users. We can mathematically write it as follows:

H_0_: *μ* = *μ*′
H_A_: *μ* > *μ*′

We applied Wilcoxon Sign-Rank test with 95% confidence interval and got a p-value < 2.57 × 10^−07^, which is much smaller than 0.05, hence, we reject the null hypothesis.

Note that the proportion of dialled numbers via contact-books was computed by assuming that if an outgoing contact was saved in the contact-book then it was assumed to be dialled through the contact-book and vice versa. In reality, there is a chance of a much higher percentage of outgoing numbers that were not dialled through the contact-book in case of emergent users. The reason is that majority of these users claimed to re-enter a number each time rather than searching in the contact list due to reading difficulty. In case of traditional users, all of them claimed to completely rely on the contact-book to redial a contact.

Now we will briefly discuss and compare the findings of all three studies with existing literature in the next section.

## Discussion

This paper explored the contact management issues of emergent users while using smartphones. Smartphones are extensively used to provide communication facility, which is mainly performed through the contact-book or call logs. Traditional textual contact-book interface is not very usable for emergent users, as these users have reading and writing difficulties. Our results are novel as we did not solely rely on the subjective opinion of users like previous studies [20, 23, 27, 54, 69], but also tried to investigate the research questions empirically which included both a usability study as well as data analysis. For example, previous studies have also explored phone-book composition and found many findings discussed here such as the use of special symbols to help emergent users recognize contacts [27], and emergent users communicating with a lesser number of people [27, 54], etc. However, we investigated these questions by data analysis, and hence these findings are more unbiased from subjective feelings of the participants than earlier studies. Moreover, the usability of contact management was not previously investigated to the best of my abilities. We will first discuss the findings corresponding to each of our research questions and we will conclude by having an overall discussion.

### Coping strategies and their effectiveness

Our first research question was to understand the coping strategies of users and their effectiveness. For this purpose, we had an interview session with our participants to understand how they use it and their expectations. We found that although the primary purpose of buying the phone was voice communication our participants used smartphones for various purposes including digital camera usage, social networks, multimedia usage, entertainment through games, music, videos, etc. This was slightly surprising because our assumption and previous research indicated that smartphones were generally purchased by economically challenged participants mainly as a status symbol [50] and their main usage was just for voice communication [13]. Our interviews also showed that our participants took help from more experienced and educated users in using their phones. We then conducted a usability study to empirically understand their problems in using the contact-book. We found that most users were unable to correctly identify both of their frequent contacts and none of them could find the infrequent numbers except one participant.

To solve the problem of contact searching, several studies suggested using icons for illiterate users, [13, 20, 76, 77]. However, this approach might not suit our user group as most of our participants indicated that recalling icons might be difficult when the number of contacts increases. Knoche et al. also observed that searching and assigning unique icons were hard tasks for such users [27]. A related approach can be hand-drawn icons [77], as the user can create a hand-drawn icon that is closer to how the contact looks. However, this will solve the problem of saving a new contact, but it would not be too helpful when searching for an existing contact. Another solution can be if users use the voice search facility, but our analysis revealed that all participants were unaware of the voice service facility for searching and saving contacts apart from one participant.

We think a more useful approach to help users in searching for existing contacts will be to use frequency and recency measurements of different contacts as discussed in various call prediction algorithms [26, 78, 79] along with a visual interface. We will present this idea in more detail in the next section.

### Composition of contact-books

We also quantitatively measured the composition of contact-books and call logs of emergent users, this was different from previous studies [20, 23, 27, 54, 69] that only considered interviews to understand this question. The results of the quantitative analysis revealed that 50% of our emergent participants had less than 50 contacts and a mean number of 94 contacts that was much smaller than the average size of traditional users’ contact-book that came out to be a mean number of 609 contacts in our analysis. Previously, Bentley et al. reported that the average size of modern contact-books of traditional users was more than 300 contacts [71]. While for emergent users, Knoche et al. [27] reported an average of 50 contacts for the participants in their study, and Joshi et al. [1] found the contact-book size between the range of 10 − 15 contacts for their participants. However, this was contrary to our results as some of our participants had no contacts saved in their contact-books due to reading writing difficulties in current textual interfaces. Similarly, the poor and illiterate users were found to have smaller social circles as compared to educated and professional people during our study. We noticed that in our emergent user sample the participants with better reading writing skills or better income resources had relatively larger contact-books. These participants included small business owners, contractors, etc.

Knoche et al. [27] reported that emergent users use the first letter of contact name followed by invented symbols to recall a contact. Our participants seemed to use a slight variation as they tended to use any random letters, digits, blank entries, and symbols, etc. to store a contact name. Our quantitative analysis of contact-books revealed that a significant proportion of 35.5% of contacts in emergent users’ contact-books was saved using such unintelligible contact names. Similarly, for the first time, we quantitatively measured that only a mean number of 35% of contacts was dialled using the contact-book or call logs by emergent users, which was much smaller than those of traditional users (i.e., 71%).

### Overall discussion

A significant decrease in the prices of handsets and low call rates encourage smartphone adoption among the low-income population. Hence, an interface design that could make the contact searching and dialling task easy for emergent users would indirectly empower illiterate persons in many aspects of their life. These people can independently search and dial the desired contacts such as their employers, co-workers, family, friends, etc., whenever needed without seeking assistance from more expert users. This would be beneficial since it is likely to increase their social activities and provide them with more opportunities to grow their businesses; as they can communicate with their clients and workers more easily and independently any time, rather than waiting for physical meetings.

Moreover, due to the worldwide COVID-19, the entire world was unable to carry out their routine tasks as normal. All social activities such as going to schools, offices, universities, hospitals, visiting family and friends were restricted. In such unavoidable circumstances that may also come again in the future, a modified contact-book could make illiterate people independent to seek assistance from the respective authorities in case of any emergency, including health issues.

Furthermore, in patriarchal societies, particularly in developing countries, women are oppressed and exploited at home by male members. They are supposed to do all the house chores and are not allowed to meet with their friends and other family members freely. Patriarchy can induce depression and anxiety in females. In such environments, a contact-book that enables illiterate women to dial the desired contact independently would provide them an opportunity to stay connected to their friends and family in their free time. This social interaction would help in improving their mental health. Similarly, women in patriarchal societies are victims of domestic violence. However, many human rights organizations and governments in developing countries have taken serious notice of women’s rights and enforce laws to provide them protection. Thus, a modified contact-book design could also empower women to fight for their rights. They can contact the respective authorities in case of any domestic violence or any other similar issue. Independent usage of phones can also be seen to empower women by allowing them to find job opportunities through their social circle. Hence, the smartphone contact-book interface serves as the interaction medium among people and it should be designed according to the needs and preference of emergent users so that they can also avail its benefits.

## Conclusion

In this paper, we investigated the contact management issues faced by emergent users in Pakistan. We hypothesized that since emergent users have difficulty in reading and writing, hence, they probably have extreme difficulty in the most basic tasks of saving and finding contacts. In this regard, we performed the following two related investigations:

- We first conducted an interview study which indicated that emergent users are indeed at a disadvantage at these basic tasks and they use several coping strategies to help them in these tasks. To confirm these findings and to understand the extent of these difficulties, we conducted a usability study to compare the performance of emergent users with the performance of traditional users in these tasks. We found that emergent users despite their coping strategies face extreme difficulties in saving and searching for a contact, including some participants unable to find a stored contact on their own phones.
- We then gathered log data of emergent users to understand their phone-book composition. We found that their phone-book had many peculiarities including assigning random numbers as names of contacts, emergent users storing very few contacts, and their preference to dial a number from scratch each time. As is obvious, these practices extremely limit their ability to use their smartphones as effectively as traditional users.

Hence, in this study, we investigate how emergent users manage their contacts. This is the limitation of this study as our findings do not directly translate into improving the usability issues we found during our investigation. Now we discuss possible future research directions. Our findings suggested some future research directions to improve the usability of contact-book and call log interfaces for emergent users. Traditional user studies have indicated that more than 80% of communication occurs with the top 10 contacts of a user [71, 79, 80] hence, we could further explore call logs of these users to look for communication proportion with top contacts. As these users already have a quite fewer number of contacts, they might have devoted more than 90% communication to these numbers. In such a situation an iconic contact-book could serve these users in a better way. However, this approach will not help them connect with more people through their phones as they still would not be able to add more than a certain number of contacts.

We think a better way to assist these users is to exploit the recent findings from call prediction [26, 78, 79, 81]. Several studies have proposed algorithms that predict who a user is going to call [78, 79] or message [26] at a given time given the user’s historical communication data. The idea is that instead of the user searching through the contact-book the users are presented a short list of contacts whom the user is more likely to call. This will make the task of users slightly easier and faster when initiating a call. Many studies indicate that users are more likely to call recent contacts, i.e. they contact with whom they recently communicated, and the contacts with whom they frequently communicate. Hence, at a given time each contact is given a recency score and a frequency score, and these scores are used to compute the most likely to be contacted contacts. Similarly, studies also indicate that users tend to communicate with their different contacts at different times of the day [81], for example, a work colleague is more likely to be contacted during work hours, while a family member or friend is more likely to be contacted during the evening hours. We think that temporal peculiarities along with frequency and recency scores can be utilized to design an improved contact-book design for emergent users. One possible solution is to display these scores and temporal information in a visual form that is easy to understand by the emergent users. Note that research in human computer interaction has shown that information presented to users in pictorial form is generally easier to understand for a user [73, 82]. We are working on the design of such an interface and we plan to evaluate how it performs on less literate users in the near future. Similarly, designing contact-books for semi-literate and illiterate users with the help of automated speech recognition can be one of such techniques that can make this interaction efficient and effective. These novel interfaces require training with different accents of local languages that could help local users for using phone-books for storing to and retrieving contacts from phone-books. In recent years sophisticated artificial intelligence systems have started to be used and they are found to influence users’ perceptions about a computing system [83–85]. In the future, we may also apply an AI-based system to assist emergent users and study how much such a system helps a user.
